# Genetic heterogeneity and clonal evolution in neuroblastoma

**DOI:** 10.1054/bjoc.2001.1849

**Published:** 2001-07

**Authors:** J Mora, N-K V Cheung, W L Gerald

**Affiliations:** Departments of Pediatrics, Pathology, Memorial Sloan-Kettering Cancer Center, New York, NY, USA

**Keywords:** heterogeneity, neuroblastoma, clonal evolution, DNA ploidy

## Abstract

Tumour heterogeneity and clonal evolution at the genetic level may explain the development of malignant or resistant disease during clinical progression of neuroblastoma (NB). In this report we use 1p allelic analysis and DNA ploidy to evaluate clonal heterogeneity and clonal selection in vivo. We studied a total of 69 tumours from 29 patients with NB. To evaluate tumour heterogeneity and clonal evolution in vivo we used a panel of polymorphic allelic markers mapping to chromosome 1. 33 tumours from 12 patients (group 1) were obtained from different sites during the same surgery or at sequential surgeries without intervening chemotherapy to evaluate genetic heterogeneity. Paired samples from 10 patients (group 2) were used to evaluate clonal selection before and after chemotherapy. In 6 cases paired tumours and derived cell lines were studied. Analysis of DNA ploidy changes by karyotype, FISH and flow cytometry was performed in 15 tumours from 6 multiply recurred local-regional (LR) NB patients. Allelotype study revealed that 66% (8/12) of group 1 samples were heterogeneous, with distinct allelic patterns in tumour samples separated by time or location. In group 2 allelic patterns were different in post-chemotherapy specimens in 60% (6/10). DNA ploidy analysis showed that pre-chemotherapy samples contained 2 distinct ploidy clones, one diploid and one triploid, whereas all post-chemotherapy tumor samples were 100% diploid. These findings suggest that NB exhibits a high degree of clonal heterogeneity and clonal evolution occurs during the course of therapy and clinical progression. © 2001 Cancer Research Campaign http://www.bjcancer.com


*British Journal of Cancer* (2001) **85**(2), 182–189
© 2001 Cancer Research Campaign
doi: 10.1054/ bjoc.2001.1849, available online at http://www.idealibrary.com on IDEAL®

### Genetic heterogeneity and clonal evolution in neuroblastoma

**J Mora[1,2], N-KV Cheung[1] and WL Gerald[2]**

[1]Departments of Pediatrics and [2]Pathology, Memorial Sloan-Kettering Cancer Center, New York, NY, USA

**Summary** Tumour heterogeneity and clonal evolution at the genetic level may explain the development of malignant or resistant disease during clinical progression of neuroblastoma (NB). In this report we use 1p allelic analysis and DNA ploidy to evaluate clonal heterogeneity and clonal selection in vivo. We studied a total of 69 tumours from 29 patients with NB. To evaluate tumour heterogeneity and clonal evolution in vivo we used a panel of polymorphic allelic markers mapping to chromosome 1. 33 tumours from 12 patients (group 1) were obtained from different sites during the same surgery or at sequential surgeries without intervening chemotherapy to evaluate genetic heterogeneity. Paired samples from 10 patients (group 2) were used to evaluate clonal selection before and after chemotherapy. In 6 cases paired tumours and derived cell lines were studied. Analysis of DNA ploidy changes by karyotype, FISH and flow cytometry was performed in 15 tumours from 6 multiply recurred local-regional (LR) NB patients. Allelotype study revealed that 66% (8/12) of group 1 samples were heterogeneous, with distinct allelic patterns in tumour samples separated by time or location. In group 2 allelic patterns were different in post-chemotherapy specimens in 60% (6/10). DNA ploidy analysis showed that pre-chemotherapy samples contained 2 distinct ploidy clones, one diploid and one triploid, whereas all post-chemotherapy tumor samples were 100% diploid. These findings suggest that NB exhibits a high degree of clonal heterogeneity and clonal evolution occurs during the course of therapy and clinical progression. © 2001 Cancer Research Campaign http://www.bjcancer.com

**Keywords**: heterogeneity; neuroblastoma; clonal evolution; DNA ploidy

Neuroblastoma (NB) is a paediatric cancer that arises from precursor cells of the peripheral sympathetic nervous system. It is clinically heterogeneous with at least 3 well recognized patterns of disease: (a) widespread disease in infants that can spontaneously regress without medical intervention; (b) local-regional (LR) disease that may recur after surgery but does not metastasize to bone or bone marrow; and (c) systemic disease with widespread marrow and skeletal metastases that initially respond to cytotoxic therapy but frequently become resistant to medical treatment. It is generally believed that such diverse clinical behaviours result from the capacity of these tumours to exploit their genetic malleability to evolve and adapt.

The study of the molecular genetics of NB in the last 2 decades has elucidated several nonrandom genetic events associated with the disease: allelic losses on chromosomes 1p, 11q, 14q, 9p, 9q, 2q, 3p, 4p and 18q implicating putative tumour suppressor genes; allelic gains on chromosomes 17q, 18q, 1q, 7 and 5q, some related to growth control genes; amplification of the oncogene *MYCN*; and changes in the normal diploid chromosomal content (Brodeur et al, 1999). The clinical role of each of these genetic events and their evolution during tumour progression are, however, largely unknown.

Prior studies have shown that NB is a genetically heterogeneous neoplasia (Brodeur, 1995). Progression or regression of the tumour may correlate with specific genetic alterations of tumour cells (Ambros et al, 1995). Chromosome 1p allelic imbalance is a common finding in NB, with patterns of LOH correlating with different categories of disease (Mora et al, 2000). These unique regions of LOH are useful clonal markers of disease progression and evolution (Mao et al, 1994). Hyperploid DNA index is a common favourable prognostic factor in LR NB and could also be used as clonal marker.

Using strict criteria for tumour quality, attention to non-neoplastic tissue contamination, microdissection to enrich tumour cell content especially in treated tumour samples, and easily quantitated fluorescent-based technology, more accurate definition of allelic imbalance among tumour subsets and their clonal evolution is possible (Larson et al, 1997). In this report we use 1p allelic analysis and DNA ploidy to evaluate clonal heterogeneity and clonal selection in vivo. Our findings suggest that genetic heterogeneity is common within each NB, and that in vivo clonal selection occurs in the presence or absence of chemotherapy.

### MATERIALS AND METHODS

### Patient materials and clinical characteristics

61 samples from 25 patients treated at MSKCC from 1987 to 1999 were studied. Cases were divided into 2 groups. Group 1 was composed of 33 tumours in 12 patients in which multiple specimens were obtained from the same patient without intervening chemotherapy and included 6 patients with specimens at different sites obtained at the same surgical procedure, and 6 patients with tumours sequentially sampled at recurrence (at diagnosis and recurrence in 4 patients, and following multiple recurrences in 2). Group 2 (25 tumours in 10 patients) consisted of paired-samples obtained at the

*Received 6 December 2000*
*Revised 12 March 2001*
*Accepted 20 March 2001*

*Correspondence to:* J Mora, E-mail: MoraJ@MSKCC.org time of diagnosis (prior to any cytotoxic therapy) and subsequently at the time of 'second-look' surgery, after the 3rd or 4th round of chemotherapy. In 6 patients, cell lines established from tumour samples were also studied.

All tumour specimens were reviewed by the same pathologist (WG) for tumour cell content and histology. All patients were staged according to the International Neuroblastoma Staging System (INSS) (Brodeur et al, 1993) and tumour histology scored according to the Shimada classification (Shimada et al, 1984). Standard treatment consisted of surgical resection for patients with local-regional disease (stages 1, 2 and 3) and the use of N6 or N7 multimodality protocols for patients with stage 4 disease as previously described (Kushner et al, 1994; Cheung et al, 1999).

### DNA content analysis

15 samples from a group of multiply recurred LR NB patients over 1 year of age were analysed for DNA ploidy. In 4 patients specimens from diagnosis and subsequent recurrences were available whereas in 2 patients only multiple relapsed post-chemotherapy specimens were available.

DNA and chromosomal content was analysed by 3 different techniques: (a) classical G-banding karyotype described according to ISCN (1985) where abnormal clones were defined as 2 or more metaphase cells with identical structural and/or numerical abnormalities, (b) interphase FISH using LSI *MYCN* DNA probe (Vysis, Inc Vysis Inc, Downers Grove, IL) and chromosome 8, 11 and 17 centromere probes to confirm aneuploidy when detected with *MYCN* probe (Grady-Leopardi et al, 1986; Shapiro et al, 1993; Taylor et al, 1994); and (c) flow cytometry (FCM) DNA analysis on nuclei isolated from paraffin sections using the method of Hedley modified (Hedley, 1989).

### Microsatellite allelic analysis

Allelic analysis was performed as previously described (Mora et al, 2000) in samples with known diploid DNA index to avoid interpretation problems from aneuploidy. Primer sequences for 12 polymorphic microsatellite loci on chromosome 1 were obtained from the Genome Data Base. Fluorescently labelled primers were obtained from Research Genetics (AL).

PCR products were initially analysed with Genescan Software (Applied Biosystems, Inc, Foster City, CA). For heterozygous samples the heights of the 2 allele peaks were used to calculate the allele ratio between the normal and tumour sample. The ratio (R) between allele peaks was calculated dividing the largest peak to the shortest peak, as recommended by the manufacturer (PE).

### RESULTS

### Clonal heterogeneity and clonal evolution of tumour DNA content

The results of the DNA and chromosomal ploidy study are summarized in Table 1. All 4 pre-chemotherapy tumour samples (cases #1, 2, 4, and 6; Table 1) available contained 2 distinct clones, one diploid and one triploid by FISH analysis of chromosomes 2, 11 and 17. Eight tumours had a triploid chromosome 2 clone detected by FISH, 3 of the 8 with DI>1, and 2 with hyperdiploid karyotypes. Histologically confirmed viable post-chemotherapy tumour samples were 100% diploid by all techniques used.

These findings suggested that heterogeneity of tumour DNA content and its evolution towards pure diploidy may be associated with multiply recurrent and lethal LR NB.

### Allelotype analysis

#### *Genetic heterogeneity and evolution in the absence of chemotherapy (Group 1)*

The existence of multiple clones within individual tumours was suggested initially by the observation of tumours showing complex patterns of allelic imbalance (AI). In some cases allelic ratios compatible with partial or subclonal loss were present in samples that exhibited complete loss of heterozygosity at adjacent loci. In other cases, ratios for one microsatellite marker varied among multiple samples from a patient when other allele ratios were maintained. By

**Table 1**

| Pt# | Tumour# | Timing | Stage | Triploid Chr. 2* % | DI | Karyotype |
|---|---|---|---|---|---|---|
| 1 | 1601 | Diagnosis | 3 | 85 | 1.55 | 48–65 XX del 1p36 |
| | 1610 | Relapse | 3 | 87 | nd | 64–70 XX del 1p36 |
| | 1868 | Post-chemo | 3 | 0 | nd | 46 XX |
| 2 | reported | Diagnosis | 1 | 35 | 1 | 46, XX, tri7 |
| | 1564 | Relapse | 3 | 0 | 1 | nd |
| 3 | 1618 | Post-chemo | 3 | 0 | 1 | 46, XY |
| | 1820 | Post-chemo | 3 | 0 | 1 | 46 XY;45 XY, +2, –3, –5 |
| 4 | 1837 | Diagnosis | 3 | 89 | 1.12 | 46, XX |
| | 1979 | Post-chemo | 4 | 0 | 1 | 46, XX |
| 5 | 1428 | Post-chemo | 3 | 75 | 1.14 | 40–45 XX |
| | 1512 | Post-chemo | 3 | 10 | nd | 44–46 XX del 1p32** |
| | 1526 | Post-chemo | 3 | 4 | nd | 46/40–45 XX |
| | 1581 | Post-chemo | 3 | 0 | nd | 44 XX |
| 6 | 1637 | Diagnosis | 3 | 72 | 1 | nd |
| | 1645 | Post-chemo | 3 | 0 | nd | 46XXdel1p22,11q13 |

*When aneuploidy was detected using LSI-MYCN probe for chromosome 2, the aneuploidy was corroborated with centromeric probes for chromosomes 8,11 and 17. **Complete karyotype: 44–46, XX, del (1)(p32), +6, add (10)(p11), +11, add(11)(q21),der(13)t(13;21) (p10;p10)x2, –16, 17, der(19)t (14;19) (q11;p13), der(21) t (21;22) (P10;P10) – 12 cells; 46, XX–8 cells.

*© 2001 Cancer Research Campaign* 

studying multiple tumour sites obtained at the same or sequential surgeries, the hypothesis of clonal heterogeneity or multi-clonality could be tested since each site may have been populated with only one or a restricted subset of tumour cell clones. The biologic, pathologic and clinical characteristics of patients in group 1 are summarized in Table 2. Of the 12 patients with multiple samples available for analysis, 8 (66%) showed different patterns of allelic ratios among their multiple tumour samples. The allelic analysis was performed at least 3 times for each discrepancy and the mean ratio of markers showing variability among samples are indicated in Table 2. The ratios in 2 cases (Cases 5, 9) are suggestive of subclonal changes in some samples with values between 0.35–0.65 indicating 30 to 60% loss or gain of one allele (see Table 2). In other samples of these same cases, the alleles were either retained (average index ratio 0.9 to 1.1) or there was more complete loss of heterozygosity demonstrated. The non-identity of LOH patterns among samples collected in the absence of selective pressure from chemotherapy – and with comparable amounts of estimated tumour cell content – suggest that allelic imbalance heterogeneity was part of the malignant process de novo.

In this group, 3 of 12 patients had heterogeneous patterns of microsatellite mutation (MM) at multiple chromosome 1 loci (Cases 1, 4, 12; Table 2). The significance of MM in these samples is not known but may reflect the instability of short tandem repeats in these tumours.

### *Clonal heterogeneity and evolution following chemotherapy (group 2)*

When paired tumour samples before and after chemotherapy were analysed, allelic discordance or clonal change following chemotherapy was present in 6 of 10 samples (Table 3). Four samples had LOH only in the pre-treatment sample (cases 2, 4, 8, 10; Table 3) with retention of heterozygosity or normalization of allelic ratio post therapy (Figure 1). Further evidence was provided by case #10 (Table 3) where deletions of markers on 1p36 identified at diagnosis were no longer detectable on the 2nd look surgery specimen. However, at the time of relapse, the original pattern of deletion was again detectable (see Figure 2). We interpret these findings to mean that clones with LOH were preferentially eliminated by chemotherapy, and might have growth advantage when compared to clones with intact alleles. However, we did not find a consistent locus of allelic change following chemotherapy that would implicate specific regions of chromosome 1 in chemosensitivity.

In 3 of the 10 pairs of samples from group 2, cases #4, 6, 10, the diagnostic and 2nd-look specimens (Table 3) had MM as evolving clonal elements. The non-germline alleles disappeared following chemotherapy in all the cases (Figure 3). Despite losing the mutated alleles in the 2nd look specimen, identical MM patterns emerged in the relapse specimen of case #10.

### *Clonal pattern and metastasis*

Loss of heterozygosity at 2 regions, 1p36 and 1p22 were associated with clinical stage in our previous study (Mora et al, 2000). To evaluate if clonal changes in vivo correlated with distant metastasis, we compared the patterns of LOH at 1p36/1p22 in 9 primary tumours versus metastases to regional lymph nodes (T#1637, 1313, 1520) or distant organs (lung: T#1635; renal hilum: T#1642; CNS: T#1596; skin: T#1437; liver: T#1840 and #877) (see Table 4). 4 showed discordant LOH in the consensus region of loss for 1p36 (D1S548 to D1S511) and 5 exhibited discordant LOH in the consensus region of loss for 1p22 (D1S481 to D1S2627). Although the number of cases analysed is small, there was no consistent change in the pattern of 1p22 or 1p36 LOH among metastatic samples.

### *Clonal pattern between primary and derived cell-lines*

In vivo tumours were compared to derived cell lines from bone marrow relapses for 6 patients (Table 5). In case #1, we analysed original tumour and recurrence, and derived cell lines from both (CL-1 and CL-2 in Table 1). Patterns of allelic imbalance differed from those of the original tumours for both cell lines. All cell lines showed more extensive regions of LOH compared to their original tumours suggesting either survival advantage or predisposition to LOH in vitro. Changes in the MM patterns were also observed with similar implications: loci of MM in the original tumour became sometimes regions of LOH while new mutations were acquired in other regions of chromosome arm 1p in derived cell lines passaged in vitro.

### DISCUSSION

Chromosome 1p LOH is a common event in NB and the pattern of losses has been correlated with clinical categories of the disease (Mora et al, 2000). The high incidence of deletions and the fact that both low and high stages of disease share this mutation, suggests that 1p LOH is an early event in the tumorigenesis of NB. Therefore LOH at 1p could be viewed as an event that confers selective advantages for continual cell replication and growth of the deleted cell clone. Changes in normal diploid DNA content are common findings among LR NB and are viewed as early events for this subgroup of NB (Mora et al, 2001). The evolution of these clones can be tracked by studying multiple tumour sites or multiple samples over time.

Prior studies have shown intratumoral genetic heterogeneity in sporadic NB cases using different biological markers including 1p LOH, *MYCN* and ploidy (Ambros et al, 1995; Brodeur, 1995; Gotoh et al, 1998). The analysis of changes in DNA ploidy over time in a selected group of relapsed LR NB showed that heterogeneity of tumour DNA ploidy and clonal evolution towards diploidy, probably related to the selective pressure of chemotherapy, was common. Furthermore the evolution towards diploidy was highly correlated with recurrence and lethality in this unusually aggressive subgroup of LR NB. In this regard, the widely held assumption, for DNA index profiles of nuclei from tumour samples that in the presence of a hyperploid peak the diploid peak reflects non-tumoral origin (Hedley, 1989), may not be true for NB. Due to the high degree of genetic heterogeneity in vivo it seems imperative to use a tumour marker besides DNA index to establish the origin of individual cells or clones.

These initial findings prompted us to a more detailed study of clonal heterogeneity in a larger series of patients with a very sensitive molecular technique. We used patterns of chromosome 1 allelic imbalance and mutation to study de novo and acquired genetic heterogeneity in individual NB samples. The use of fluorescent technology to analyse microsatellite sequences has made it easier to identify and quantitate allele bands (Cawkwell et al, 1993; Larson et al, 1997) and provides considerable advantages over conventional approaches: (a) it allows the visualization of alleles using an electropherogram which simplifies its recognition; and (b) uses an internal standard based on size allowing a precise evaluation of repeat length differences. Using this technology we analysed over 3000 genotypes for LOH on chromosome 1 of 180

 © 2001 Cancer Research Campaign

**Table 2** Comparison of tumours collected from different sites or times (group 1)

| Pt# | Sample | Site | TCC | STAGE | PLOIDY | *MYC-N* | 1p36.3 D1S548 | 35 D1S592 | 35 D1S552 | 34.1 D1S511 | 31 MycL | 31 D1S1669 | 22 D1S1596 | 22 D1S2766 | 22 D1S1618 | 21 D1S1673 | 1qtel D1S1602 | 1qtel D1S547 |
|---|---|---|---|---|---|---|---|---|---|---|---|---|---|---|---|---|---|---|
| 1 | 755-Dx | retroperitoneum | 100% | LR (2) | 1 | NA | 1.64 | H | 3.4 | H | 1.05 | 1.1 | MM | H | | 1.1 | + | H |
| | 1252-rel | retroperitoneum | 80% | | | | 1.76 | H | 0.9 | H | 1.2 | | – | H | + | 1.6 | + | H |
| 2 | 1386-Dx | adrenal T. | 100% | 4 | 1 | A | H | H | – | H | – | H | + | + | – | H | + | + |
| | 1635-rel | lung T. | 90% | | | | H | H | – | H | – | H | + | + | – | H | + | + |
| 3 | 1631-Dx | adrenal T. | 80% | 4 | 1 | NA | + | H | + | H | + | + | + | + | + | + | + | + |
| | 1642-rel | renal hilium T. | 80% | | | | + | H | + | H | + | + | + | + | + | + | + | + |
| | 1896-rel | | 80% | | | | + | H | + | MM | MM | + | + | + | | + | + | |
| 4 | 1369-Dx | adrenal T. | 70% | 4 | 1 | NA | MM | + | H | 0.1 | MM | MM | 1 | MM | MM | – | + | MM |
| | 1596-rel | CNS T. | 80% | | | | MM | + | H | 1 | + | MM | 0.1 | H | – | – | + | + |
| 5 | 1428-rel | colon T. | 80% | LR (3) | 1.1 | NA | H | H | + | + | + | + | + | 0.2 | MM | + | + | + |
| | 1439-rel | pelvic T. | 70% | | | | H | H | + | + | 0.5 | + | + | 0.2 | MM | + | + | + |
| | 1512-rel | pelvic T. | 70% | | | | H | H | + | + | 1 | + | + | 1 | MM | + | + | + |
| | 1526-rel | retroperitoneal T. | 70% | | | | H | H | | + | 1.2 | + | + | 1 | 0.2 | + | + | + |
| | 1563-rel | retroperitoneal T | 90% | | | | H | H | + | + | 1.03 | + | + | 1 | + | + | + | + |
| | 1581-rel | retroperitoneal T | 90% | | | | H | H | + | + | 1.2 | + | + | 1 | MM | + | + | + |
| 6 | 1127-rel | neck LN | 100% | 4n | 1 | NA | + | + | H | H | – | – | 2 | + | – | H | + | H |
| | 1397-rel | neck LN | 100% | | | | + | + | H | H | – | – | 1.1 | + | – | H | + | H |
| | 96-rel | neck LN | 100% | | | | + | + | H | H | – | – | 1 | + | – | H | + | H |
| 7 | 1637-Dx | cervical LN | 70% | LR (3) | 1 | NA | H | + | + | + | + | H | + | + | H | H | + | |
| | 1643-Dx | epidural T | 60% | | | | H | + | + | 0.8 | + | H | + | + | H | H | + | – |
| 8 | 1377-Dx | adrenal T. | 100% | 4 | 1.04 | A | + | + | H | + | + | + | + | + | 1.4 | + | + | + |
| | 1437-Dx | skin abdomen | 80% | | | NA | + | + | H | + | + | + | + | + | 1.6 | + | + | + |
| 9 | 1312-Dx | mediastinal T. | 100% | 4 | 1 | NA | + | – | – | – | H | + | + | – | H | 1.5 | 2 | H |
| | 1313-Dx | Richest wall LN | 100% | | | | + | – | – | – | H | + | + | – | MM | 1.6 | 2 | H |
| | 1314-Dx | Diaphragma LN | 100% | | | | + | – | – | – | H | + | + | | | 1.1 | 1 | H |
| | 1315-Dx | Lichest wall LN | 100% | | | | + | – | – | – | H | + | + | | | 1.6 | 2 | H |
| 10 | 1841-Dx | Adrenal T. | 90% | 4 | 1 | NA | – | 2 | 0.3 | H | + | 0.7 | 0.8 | + | – | – | + | + |
| | 1840-Dx | liver | 80% | | | | – | 0.9 | 0.9 | H | + | 1.9 | 1.8 | – | – | – | + | + |
| 11 | 1434-2nd | spinal T. | 70% | 4 | 1 | NA | – | + | + | + | + | H | + | + | H | – | H | + |
| | 1435-2nd | primary T. | 80% | | | | – | + | + | + | + | H | + | + | H | – | H | + |
| | 1520-2nd | axillary LN | 80% | | | | – | + | + | + | + | H | + | + | H | – | H | + |
| 12 | 877-rel | Liver nodule | 70% | 4 | 1 | A | – | MM | 1 | – | + | MM | MM | MM | + | MM | H | MM |
| | 878-rel | adrenal T. | 50% | | | A | – | – | 0.1 | – | + | MM | MM | MM | + | MM | H | MM |

Dx=sample at diagnosis; rel = sample at relapse; 2nd = sample taken during active course of chemotherapy. T = tumour; LN = lymph node; TCC = tumour cell content;: LR = local-regional disease (INSS stages 1,2 or 3). 4 = INSS stage 4; 4n = INSS stage 4n; NA = non amplified; A = amplified. + = retained heterozygosity; – = LOH; H = non informative; MM = microsatellite mutation; Discordances are shaded grey. Numbers in the table are allelic ratios for discordant samples.

© 2001 Cancer Research Campaign

**Table 3** Comparison of tumours before and after chemotherapy (Group 2)

| Pt# | Sample | Site | TCC | STAGE | PLOIDY | *MYC–N* | 1p36.3 D1S548 | 35 D1S1592 | 35 D1S552 | 34.1 D1S511 | 31 MycL | 31 D1S1669 | 22 D1S1596 | 22 D1S2766 | 22 D1S1616 | 21 D1S1673 | 1qtel D1S1602 | 1qtel D1S547 |
|---|---|---|---|---|---|---|---|---|---|---|---|---|---|---|---|---|---|---|
| 1 | 1129–Dx | liver | 90% | 4 | 1 | NA | H | + | + | + | – | + | H | | | | + | H |
| | 1147–2nd | adrenal T. | 80% | | | | H | + | + | + | – | + | H | + | + | + | + | H |
| 2 | 1012–Dx | portal LN | 75% | 4 | 1 | A | H | H | H | H | 0.1 | 0.1 | 0.1 | 0.1 | H | 0.1 | 0.1 | H |
| | 1035–2nd | adrenal T. | 75% | | | | H | H | H | H | 1 | 1 | 1 | 1 | H | 1 | 1 | H |
| 3 | 1419–Dx | abdominal LN | 90% | 4 | 1 | NA | H | + | + | + | MM | + | H | + | 1 | + | MM | + |
| | 1466–2nd | retroperitoneal T | 70% | | | | H | + | + | + | + | + | H | + | 0.1 | + | + | + |
| 4 | 1452–Dx | adrenal T. | | 4 | 1 | NA | H | H | + | + | H | + | + | + | 0.1 | + | + | + |
| | 1523–2nd | adrenal T. | 100% | | | | H | H | + | + | H | + | + | + | 1 | + | + | + |
| | 1524–2nd | pelvic T | 90% | | | | H | H | + | + | H | + | + | + | 1 | + | + | + |
| 5 | 1828–Dx | mediastinal T | 75% | 4 | 1 | NA | + | + | + | + | + | H | H | + | H | + | H | + |
| | 1855–2nd | mediastinal T | 100% | | | | + | + | + | + | + | H | H | + | H | + | H | + |
| 6 | 1647–Dx | abdominal T | 70% | 4 | 1 | NA | MM | + | MM | H | MM | MM | MM | 1 | 0.1 | MM | MM | 1.6 |
| | 1844–2nd | abdominal T | 80% | | | | H | + | + | H | + | + | + | 0.3 | 1 | + | + | 1.2 |
| | 1845–2nd | abdominal T | 70% | | | | H | + | + | H | + | + | + | – | 1 | + | + | 1.1 |
| 7 | 1827–Dx | retroperitoneal T. | 80% | 4 | 1 | NA | H | H | + | + | + | + | + | + | + | + | + | + |
| | 1852–2nd | adrenal T. | 90% | | | | H | H | + | + | + | + | + | + | + | + | + | + |
| | 1853–2nd | celiac LN | 70% | | | | H | H | + | + | + | + | | + | + | + | + | + |
| 8 | 1267–rel | pandreatic T. | 60% | 4 | 1 | NA | H | H | H | + | + | + | 0.1 | + | H | 1 | + | + |
| | 1270–2nd | liver | 70% | | | | H | H | H | + | + | + | 1 | + | H | 1 | + | + |
| | 1623–rel | cervical T. | | | | | H | H | H | + | + | – | 1 | + | H | 0.2 | + | + |
| 9 | 1517–rel | retroperitoneal T. | 70% | 4 | 1 | A | H | – | – | – | – | + | + | + | + | + | – | + |
| | 1565–2nd | retroperitoneal T. | 80% | | | | H | – | – | – | – | + | + | + | + | + | – | + |
| 10 | 1130–Dx | inguinal LN | 70% | 4 | 1 | NA | + | 0.3 | 0.4 | MM | MM | 0.9 | MM | 0.3 | + | 0.6 | 0.4 | MM |
| | 1170–2nd | adrenal T. | | | | | + | 0.8 | 0.7 | + | + | 0.9 | H | 0.6 | + | 0.8 | 0.8 | + |
| | 1436–rel | paraspinal T. | 50% | | | | + | 0.2 | 0.2 | MM | MM | 0.3 | MM | 0.1 | – | 0.4 | 0.9 | MM |

Dx = sample at diagnosis; rel = sample at relapse; 2nd = sample taken during active course of chemotherapy. T = tumour, LN = lymph node; TCC = tumour cell content;: LR = local–regional disease (INSS stages 1,2 or 3). 4 = INSS stage 4; 4n = INSS stage 4n; NA = non amplified; A = amplified. +=retained heterozygosity; – = LOH; H = non informative; MM = microsatellite mutation; Discordances are shaded grey. Numbers in the table are allelic ratios for discordant samples.

© 2001 Cancer Research Campaign

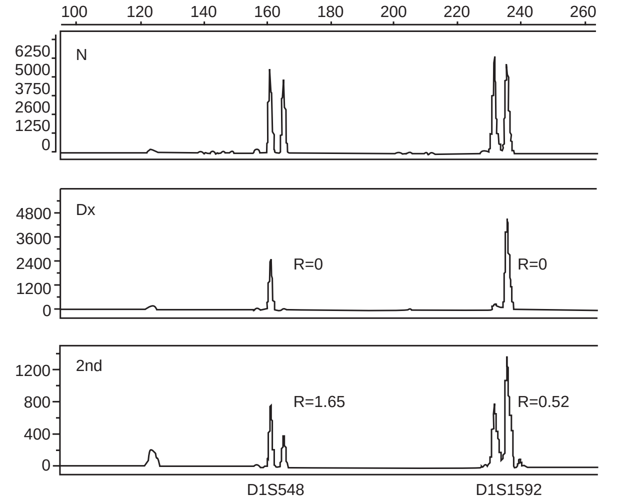

**Figure 1** Representative electropherograms. N is analysis of DNA from normal tissue and Dx/2nd is from tumour samples taken at diagnosis or at second-look surgery respectively. There is LOH for marker D1S548 at 1p36 with ratio close to 0 in the sample from diagnosis and 1.65 in the second-look surgery sample and a nearby marker, D1S1592 on 1p36, shows LOH with a ratio close to 0 in the diagnostic specimen and 0.52 in the second-look specimen

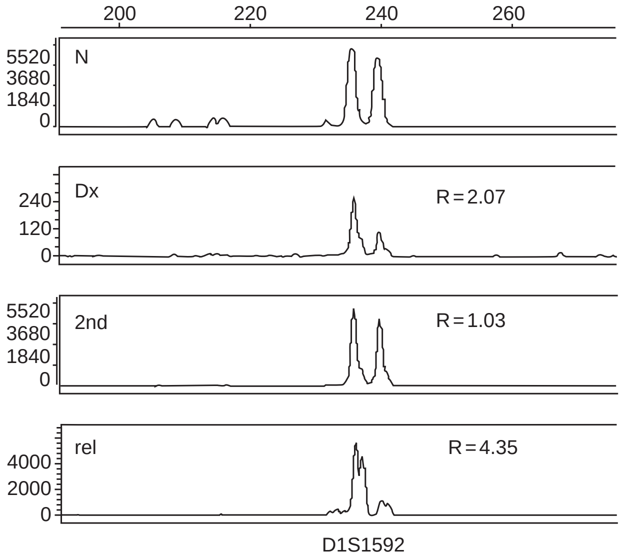

**Figure 2** Representative electropherograms for Case #10, Table 3. N is analysis of DNA from normal tissue and Dx/2$^{nd}$/rel is from tumour samples taken at diagnosis, at second-look surgery and at relapse respectively. The analysis of D1S1592 marker on chromosome 1p36 is shown. The ratios for each tumour sample compared to the normal tissue show LOH at diagnosis (2.07) and relapse (4.35) specimens and retention of heterozygosity ratio (1.03) for the second-look surgery specimen

NB tumours from 120 patients (Mora et al, 2000). During this analysis MM phenotype defined as mutations affecting more than 30% of markers was identified in 20 (16%) of the 120 NB patients. Further characterization established that mutations within this group were more frequent in tri-and tetra-nucleotide repeats, and occurred at multiple loci, but was most often detected in chromosomal regions previously identified as frequently loss in NB. MM tumours were also analysed with 12 microsatellite markers mapping to 4 different chromosomes. A consistent pattern of more than 30% of markers mutated was observed all throughout the

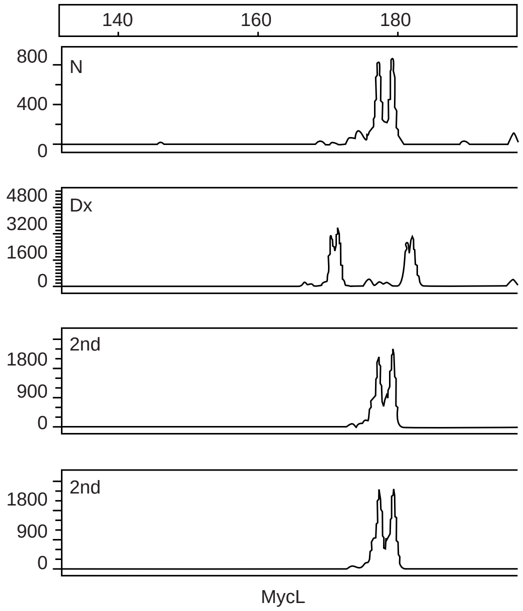

**Figure 3** Representative electropherograms for Case #6, Table 3. N is analysis of DNA from normal tissue and Dx/2nd is from tumour samples taken at diagnosis and second-look surgery respectively. The analysis of MycL marker on chromosome 1p31 is shown. MM is detected for MycL only in the diagnostic sample compared to normal pattern and 2nd-look surgery specimen patterns genome regardless of the type of marker (di-, tri- or tetra-nucleotide) or the chromosome location. In all cases there was at least 30% preservation of other polymorphic alleles for matched samples. Our somewhat higher incidence of MM compared to previous reports in NB (Martinsson et al, 1995) may be explained by increased sensitivity and the use of more tetranucleotide repeat markers compared to prior studies. After amplification by PCR, each allele is detected as a predictable component of a complex signature that can be followed over time or among multiple tumour sites. We analysed a series of patients with tumour samples of known diploid DNA content to avoid interpretation problems. Samples were collected over time to follow spontaneous or therapy related molecular evolution and from different sites to study the clonal composition of metastatic foci. We found intra-tumoral heterogeneity using chromosome 1 AI and MM patterns to be common. For these patients individual tumour samples were genetically related but not identical, and clonal populations spontaneously evolved during tumour progression.

Although we found intratumor clonal heterogeneity and clonal evolution in vivo over time, we did not detect a consistent pattern of accumulation of chromosome 1p changes in the evolution of multiply relapsed specimens in the absence of therapy. This suggests 1p LOH is not a mutation driving the progression of tumours with acquisition of more aggressive phenotypes in late stages of disease. In vitro cell lines established from tumours however showed larger and more widespread 1p mutations. These findings suggest that in vitro selective pressure may differ significantly from in vivo selective pressures such as microenvironment, immune surveillance and genetic background.

Our data suggest that clonal genetic populations can be selected for by cytotoxic therapy. The disappearance of aneuploid clones and clones with 1p LOH during chemotherapy, and in one case the reappearance of the same 1p-deleted clones at relapse, would suggest that in this case 1p-deleted clones were

*© 2001 Cancer Research Campaign*

**Table 4** Comparison of primary tumour and distant metastases

| Pt# | Sample | Primary/distant organ | TCC | 1p36.3 D1S548 | 35 D1S1592 | 35 D1S552 | 34.1 D1S511 | 22 D1S481 | 22 D1S2766 | 22 D1S1618 | 21 D1S1673 | 21 D1S2627 |
|---|---|---|---|---|---|---|---|---|---|---|---|---|
| 1 | 1386–Dx | adrenal T. | 100% | H | H | – | H | + | + | – | H | + |
| | 1635–rel | lung T. | 90% | H | H | – | H | + | + | – | H | + |
| 2 | 1631–Dx | adrenal T. | 80% | + | H | + | H | + | + | + | + | + |
| | 1642–rel | renal hilium T. | 80% | + | H | + | H | + | + | + | + | + |
| 3 | 1369–Dx | adrenal T. | 70% | MM | + | H | – | + | MM | MM | – | MM |
| | 1596–rel | CNS T. | 80% | MM | + | H | + | – | H | – | – | H |
| 4 | 1377–Dx | adrenal T. | 100% | + | + | H | + | + | + | + | + | MM |
| | 1437–prg | skin abdomen | 80% | + | + | H | + | + | + | – | + | MM |
| 5 | 1841–Dx | Adrenal T. | 90% | – | – | – | H | + | + | – | – | – |
| | 1840–prg | liver | 80% | – | + | + | H | + | – | – | – | – |
| 6 | 878–rel | adrenal T. | 50% | – | – | – | – | – | MM | + | MM | + |
| | 877–rel | Liver nodule | 70% | – | MM | + | – | – | MM | + | MM | + |
| | | Primary/regional LN | | | | | | | | | | |
| 7 | 1643–Dx | epidural T | 60% | H | + | + | + | + | + | H | H | + |
| | 1637–Dx | cervical LN | 70% | H | + | + | + | + | + | H | H | + |
| 8 | 1312–Dx | mediastinal T. | 100% | + | – | – | – | H | – | H | – | + |
| | 1313–Dx | R/chest wall LN | 100% | + | – | – | – | H | – | MM | – | + |
| 9 | 1435–2nd | primary T. | 80% | – | + | + | + | H | + | H | – | + |
| | 1520–2nd | axillary LN | 80% | – | + | + | + | H | + | H | – | + |

Dx = sample at diagnosis; rel = sample at relapse; 2nd = sample taken during active course of chemotherapy. T = tumour; LN = lymph node; TCC = tumour cell content;: LR = local–regional disease (INSS stages 1,2 or 3). 4 = INSS stage 4; 4n = INSS stage 4n; NA = non amplified; A = amplified. + = retained heterozygosity; – = LOH; H = non informative; MM = microsatellite mutation; Discordances are shaded grey. Numbers in the table are allelic ratios for discordant samples.

**Table 5** Comparison of in vivo tumour sample with derived cell lines

| Pt# | Sample | 1p36.3 D1S548 | 1p35 D1S1592 | 1p35 D1S552 | 1p34.1 D1S511 | 1p31 MycL | 1p31 D1S1669 | 1p22 D1S1596 | 1p22 D1S481 | 1p22 D1S2766 | 1p21 D1S1673 | 1qtel D1S1602 | 1qtel D1S547 |
|---|---|---|---|---|---|---|---|---|---|---|---|---|---|
| 1 | 755–Dx | 1.6 | H | 3.4 | H | 1.05 | 1.17 | MM | + | H | 1.1 | + | H |
| | 1252–rel | 1.7 | H | 0.9 | H | 1.2 | | – | + | H | 1.6 | + | H |
| | CL–1 | 1 | H | 0.9 | H | 1.2 | 1.1 | – | + | | 1.7 | + | H |
| | CL–2 | MM | MM | 0.1 | H | 0.1 | 0.1 | – | 0.1 | | MM | MM | MM |
| 2 | 1386–Dx | H | H | 0.1 | H | 0.2 | H | + | + | + | H | + | + |
| | 1635–rel | H | H | 0.2 | H | 0.3 | H | + | + | + | H | + | + |
| | CL | H | H | | | MM | H | + | + | + | | MM | |
| 3 | 1428–rel 1 | H | H | + | + | + | + | + | + | 0.2 | + | + | + |
| | 1439–rel 2 | H | H | + | + | 0.5 | + | + | + | 0.2 | + | + | + |
| | 1512–rel 3 | H | H | + | + | 1 | + | + | + | 1 | + | + | + |
| | 1526–rel 4 | H | H | | + | 1.2 | + | + | + | 1 | + | + | + |
| | 1563–rel 5 | H | H | + | + | 1.03 | + | + | + | 1 | + | + | + |
| | 1581–rel 6 | H | H | + | + | 1.2 | + | + | + | 1 | + | + | + |
| | CL | H | MM | + | MM | 0.1 | 0.1 | 0.1 | + | + | 0.1 | 0.1 | + |
| 4 | 827–Dx | 0.1 | 0.2 | MM | MM | + | MM | MM | 0.2 | MM | + | 0.2 | MM |
| | CL | 0.1 | 0.2 | 0.1 | MM | 0.1 | MM | | 0.1 | MM | + | MM | MM |
| 5 | 1876–Dx | | H | H | 0.1 | 0.1 | 0.1 | 0.1 | + | 0.2 | 0.1 | + | H |
| | CL | H | H | | 0.2 | 0.3 | 0.2 | 0.2 | + | 0.1 | 0.1 | + | H |
| 6 | 1172–rel | + | + | + | + | + | + | + | H | + | + | + | H |
| | CL | 0.1 | 0.1 | 0.1 | 0.1 | 0.1 | MM | MM | + | MM | 0.1 | + | MM |

Dx = sample at diagnosis; rel = sample at relapse; 2nd = sample taken during active course of chemotherapy. T = tumour; LN = lymph node; TCC = tumour cell content; LR = local–regional disease (INSS stages 1,2 or 3). 4 = INSS stage 4; 4n = INSS stage 4n; NA = non amplified; A = amplified. += retained heterozygosity; –= LOH; H = non informative; MM = microsatellite mutation; Discordances are shaded grey. Numbers in the table are allelic ratios for discordant samples.

more sensitive to cytotoxic treatments than 1p-intact ones. Differential proliferative rates may explain their different chemosensitivity. Whether new 1p deletions were acquired at relapse, or whether the original 1p-deleted clones regrew during clinical relapse is an open and important question. A better understanding of the evolution of such subclones and their

 *© 2001 Cancer Research Campaign*

response to treatment may be critical in the future design of curative strategies. The molecular characterization of these tumour clones, and their correlation with histologic subtypes may provide clinical insights in the molecular staging and prognostication of neuroblastoma.

The accumulation of genetic abnormalities has been suggested as a mechanism for the development of metastatic capability (Fearon and Vogelstein, 1990). Recent reports on NB have identified differing biological features at primary and metastatic sites, implying clonal selection for some biological markers in metastasis (Suzuki et al, 1997; Gotoh et al, 1998). In our study, the comparison of 1p LOH patterns between original tumour sites and metastasis in lymph nodes or distant organs did not reveal any preferential 1p loss. Although clonal heterogeneity was common between sites, we did not find a consistent increase in number or size of deletions in metastatic samples. Other studies have shown similarly an absence of changes in *MYCN* amplification or ploidy between primary and metastatic sites (Brodeur et al, 1987; Taylor et al, 1988). Our results would suggest that the ability of tumours to metastasize is determined by genetic features other than 1p LOH.

Since non-progressing NB tumours were not analysed because of the lack of material and evolution, the data presented demonstrates that intratumoral clonal heterogeneity is a common part of the malignant process de novo in progressing NB tumours. More importantly, there appears to be genetic selection during cytotoxic therapy that may reflect differences in chemosensitivity among the different clones. Such information might be useful for predicting response and tailoring therapy for individual patients.

### ACKNOWLEDGEMENTS

Supported in part by the Robert Steel Foundation, New York, NY, the Katie's Find A Cure Fund, New York, NY, the Justin Zahn Fund, New York, NY. J.M. is the recipient of an ASCO 2000 YIA.

### REFERENCES

Ambros PF, Ambros IM, Strehl S, Bauer S, Luegmayr A, Kovar H, Ladenstein R, Fink FM, Horcher E, Printz G, Mutz I, Schilling F, Urban C and Gadner H (1995) Regression and progression in neuroblastoma. Does genetics predict tumor behaviour? *Eur J Cancer* **31A**: 510–515

Ambros IM, Zellner A, Roald B, Amann G, Ladenstein R, Printz D, Gadner H and Ambros PF (1996) Role of ploidy, chromosome 1p, and Schwann cells in the maturation of neuroblastoma. *N Engl J Med* **334**: 1505–1511

Brodeur GM (1995) Molecular basis for heterogeneity in human neuroblastomas. *Eur J Cancer* **31A**: 505–510

Brodeur GM, Hayes FA, Green AA, Casper JT, Wasson J, Wallach S and Seeger RC (1987) Consistent N-myc copy number in simultaneous or consecutive neuroblastoma samples from 60 individual patients. *Cancer Res* **47**: 4248–4253

Brodeur GM, Pritchard J, Berthold F, Carlsen NLT, Castel V, Castleberry RP, De Bernardi B, Evans AE, Favrot M, Hedborg F, Kaneko M, Kemshead J, Lee REJ, Look T, Pearson ADJ, Philip T, Roald B, Sawada T, Seeger RC, Tsuchida Y and Voute PA (1993) Revisions of the International Criteria for neuroblastoma diagnosis, staging, and response to treatment. *J Clin Oncol* **11**: 1466–1477

Brodeur GM, Sawada T, Tsuchida Y, Voute PA (1999) Neuroblastoma. Elsevier Science: Amsterdam

Cawkwell L, Bell SM, Lewis FA, Dixon MF, Taylor GR, and Quirke P (1993) Rapid detection of allele loss in colorectal tumours using microsatellites and fluorescent DNA technology. *Br J Cancer* **67**: 1262–1267

Cheung NKV, Kushner BH, LaQuaglia M, Kramer K, Gollamudi S, Heller G, Gerald W, Yeh S, Finn R, Larson SM, Wuest D, Byrnes M, Dantis E, Mora J, Cheung IY, Rosenfield N, Abramson S, and O'Reilly RJ (2001) N7 a novel multi-modality therapy of high risk neuroblastoma in children diagnosed over 1 year of age. *Med and Ped Oncol* **36**: 227–230

Fearon ER and Vogelstein B (1990) A genetic model for colorectal tumorigenesis. *Cell* **61**: 759–767

Gotoh T, Sugihara H, Matsumura T, Katsura K, Takamatsu T and Sawada T (1998) Human neuroblastoma demonstrating clonal evolution in vivo. *Genes Chromosomes Cancer* **22**: 42–49

Hedley DW (1989) Flow cytometry using paraffin-embedded tissue: five years on. *Cytometry* **10**: 229–241

Kushner BH, LaQuaglia MP, Bonilla MA, Lindsley K, Rosenfield N, Yeh S, Eddy J, Gerald WL, Heller G and Cheung NKV (1994) Highly effective induction therapy for stage 4 neuroblastoma in children over 1 year of age. *J Clin Oncol* **12**: 2607–2613

Larson AA, Kern S, Curtiss S, Gordon R, Cavenee WK and Hampton GM (1997) High resolution analysis of chromosome 3p alterations in cervical carcinoma. *Cancer Res* **57**: 4082–4090

Mao L, Lee DJ, Tockman MS, Erozan YS, Askin F and Sidransky D (1994) Microsatellite alterations as clonal markers for the detection of human cancer. *Proc Natl Acad Sci* **91**: 9871–9875

Martinnsson T, Sjoberg RM, Hedborg F et al (1995) Deletion of chromosome 1p loci and microsatellite instability in neuroblastomas analyzed with short-tandem repeat polymorphisms. *Cancer Res* **55**: 5681–5686

Mora J, Cheung NKV, Kushner BH, LaQuaglia MP, Kramer K, Fazzari M, Heller G, Chen L and Gerald W (2000) Clinical Categories of Neuroblastoma are associated with different Patterns of Loss of Heterozygosity on Chromosome Arm 1p. *J Mol Diagn*, **2**: 37–46

Mora J, Gerald W, Chen L, Qin J and Cheung NKV (2001) Survival analysis of clinical, pathologic and genetic features in neuroblastoma presenting as local-regional disease. *Cancer* **91**: 435–442

Shimada H, Chatten J, Mewton WA, Sachs N, Hamoudi AB, Chiba T, Marsden HB and Misugi K (1984) Histopathologic prognostic factors in neuroblastic tumors: definition of subtypes of ganglioneuroblastoma and an age-linked classification of neuroblastomas. *J Natl Cancer Inst* **73**: 405–416

Shimada H, Ambros IM, Dehner LP, Hata J, Joshi VV and Roald B (1999) Terminology and morphologic criteria of neuroblastic tumors: recommendations by the International Neuroblastoma Pathology Committee. *Cancer* **86**: 349–363

Suzuki T, Mugishima H, Chin M, Takamura M, Shichino H, Nagata T and Harada K (1997) Case of neuroblastoma with differing cytologic and molecular biologic features at primary and metastatic sites. *J Ped Hem/Onc* **19**: 176–180

Taylor SR, Blatt J, Constatino JP, Roederer M and Murphy RF (1988) Flow cytometric DNA analysis of neuroblastoma and ganglioneuroma. *Cancer* **62**: 749–754

© 2001 Cancer Research Campaign 
